# Supplementary material for: Mechanically resilient hybrid aerogels containing fibers of dual-scale sizes and knotty networks for tissue regeneration
Source: Nat Commun. 2024 Feb 5;15:1080. doi: 10.1038/s41467-024-45458-x (PMC10844217; doi:10.1038/s41467-024-45458-x)
Supplement: Supplementary file 1 — Supplementary Information [file 41467_2024_45458_MOESM1_ESM.pdf]

Supplementary Information

Mechanically resilient hybrid aerogels containing fibers with dual-scale sizes and knotty networks for tissue regeneration

S. M. Shatil Shahriar[1,2], Alec D McCarthy[1], Syed Muntazir Andrabi[1], Yajuan Su[1], Navatha Shree Polavoram[1], Johnson V. John[1], Mitchell P. Matis[1], Wuqiang Zhu[3], and Jingwei Xie[1,4]*

[1]Department of Surgery – Transplant and Mary & Dick Holland Regenerative Medicine Program, College of Medicine, University of Nebraska Medical Center, Omaha, NE, 68198, USA

[2]Eppley Institute for Research in Cancer and Allied Diseases, College of Medicine, University of Nebraska Medical Center, Omaha, NE, 68198, USA

[3]Department of Cardiovascular Diseases, Physiology and Biomedical Engineering, Center for Regenerative Medicine, Mayo Clinic Arizona, Scottsdale, AZ, 85259, USA

[4]Department of Mechanical and Materials Engineering, University of Nebraska Lincoln, Lincoln, NE, 68588, USA

[*]e-mail address: jingwei.xie@unmc.edu (J. Xie)

Table S1. Summary of mechanical properties.

Criteria	Weight	NFA	MFA	NF/MF-A1	NF/MF-A2	NF/MF-A3
Compressive Strength (MPA)	0.13	1.802	6.472	28.758	15.311	23.715
Tearing Strength (N)	0.18	2.466	4.678	14.816	9.837	12.167
Fracture Energy (kJ/m^2)	0.14	1.233	2.339	7.408	4.918	6.083
Youngs modulus (MPA)	0.15	3.57	15.327	75.482	30.807	56.044
Toughness (KJ/m^3)	0.3	92.782	398.263	1961.367	800.509	1456.292
Density (g/cm^3)	0.1	0.036	0.064	0.041	0.059	0.053
Total		101.889	427.143	2087.872	861.441	1554.354
Weighted Average		29.225	123.795	607.179	249.229	451.424

Table S2. Weighted T-Score table.

**Criteria**	**Weight**	**NFA**	**MFA**	**NF/MF-A1**	**NF/MF-A2**	**NF/MF-A3**
Compressive Strength (MPa)	0.13	51.11	62.42	64.112	57.54	63.88
Tearing Strength (N)	0.18	59.27	58.89	63.92	58.333	60.52
Fracture Energy (kJ/m^2)	0.14	53.57	58.57	62.49	52.5	58.57
Young's modulus (MPa)	0.15	53.33	55.66	60.00	56.54	59.62
Toughness (KJ/m^3)	0.3	58.88	62.76	78.42	63.56	68.90
Density (g/cm^3)	0.1	58.06	58.64	66.14	59.86	63.02
Total		334.25	356.94	395.08	348.33	374.00
Weighted Average		56.28	59.95	67.72	60.96	63.31

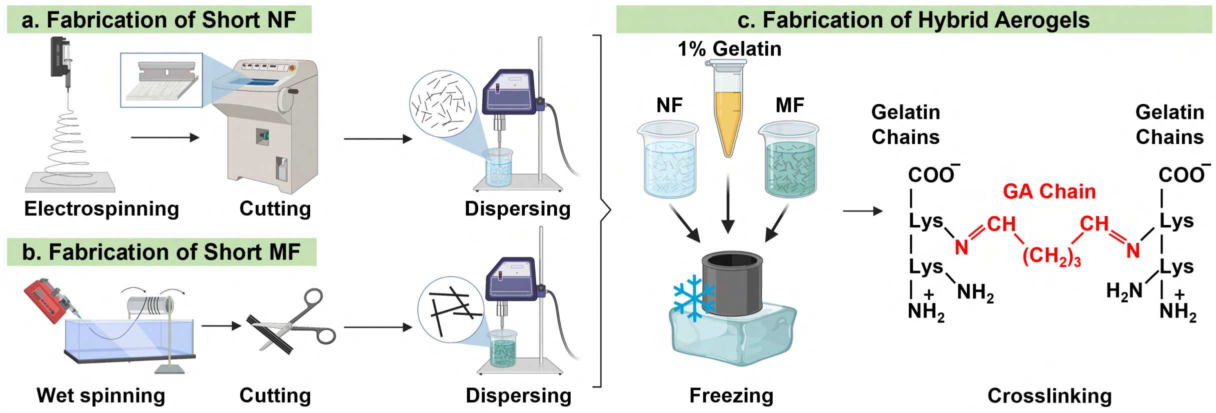

Figure S1 | Schematic showing the fabrication of 3D hybrid aerogels. a, Preparation of short NF: producing NF mats by electrospinning, cryo-cutting 2D NF mats into short NFs, treating plasma, and dispersing NFs. **b**, Fabrication of short MF: producing MFs by wet spinning, cutting them into short MFs, treating plasma, and dispersing MFs. **c**, Fabrication of hybrid aerogels: freeze-drying of the NF and MF suspension containing gelatin as a crosslinker in a 3D-printed mold followed by crosslinking with GA vapor and removal of the mold. The schematic was created in BioRender.com.

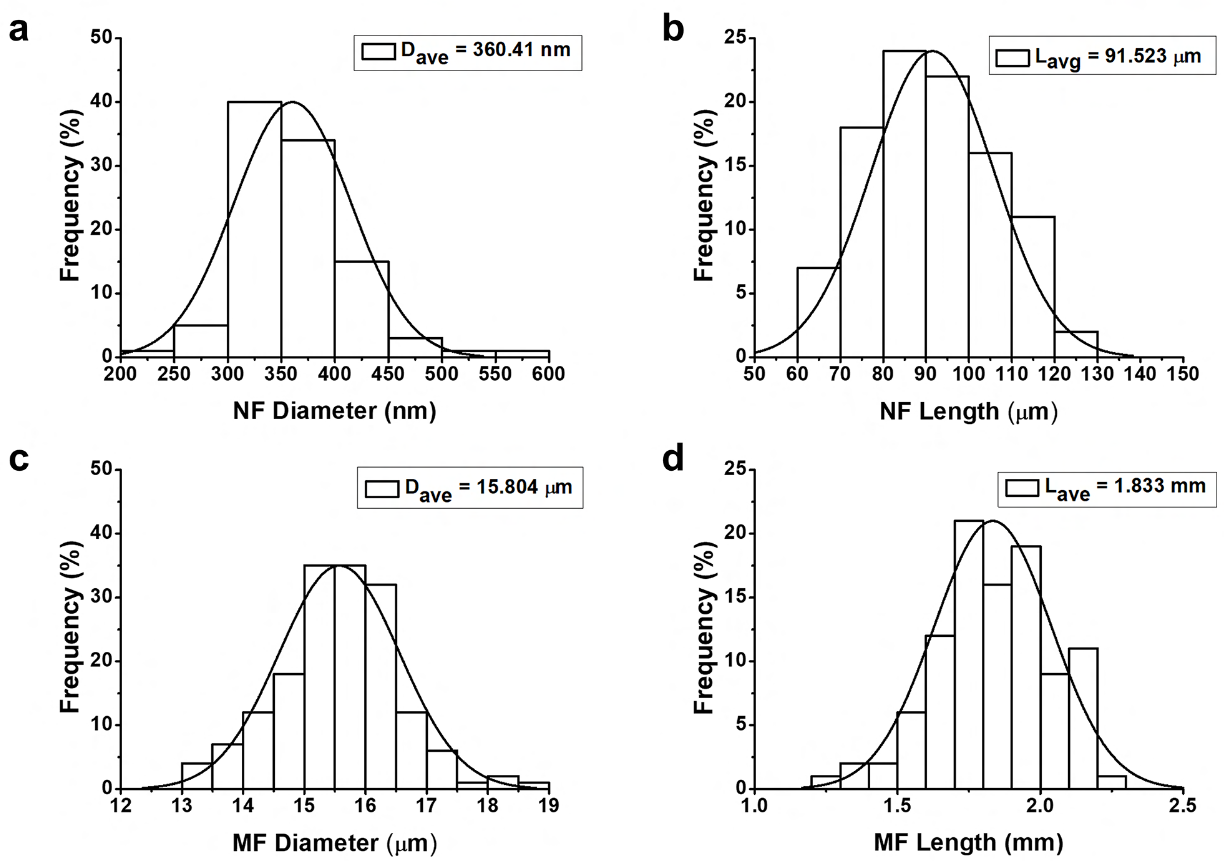

Figure S2 | Characterization of short NFs and MFs. a,**b**, Distributions of diameter and length of short NFs. **c**,**d**, Distributions of diameter and length of short MFs.

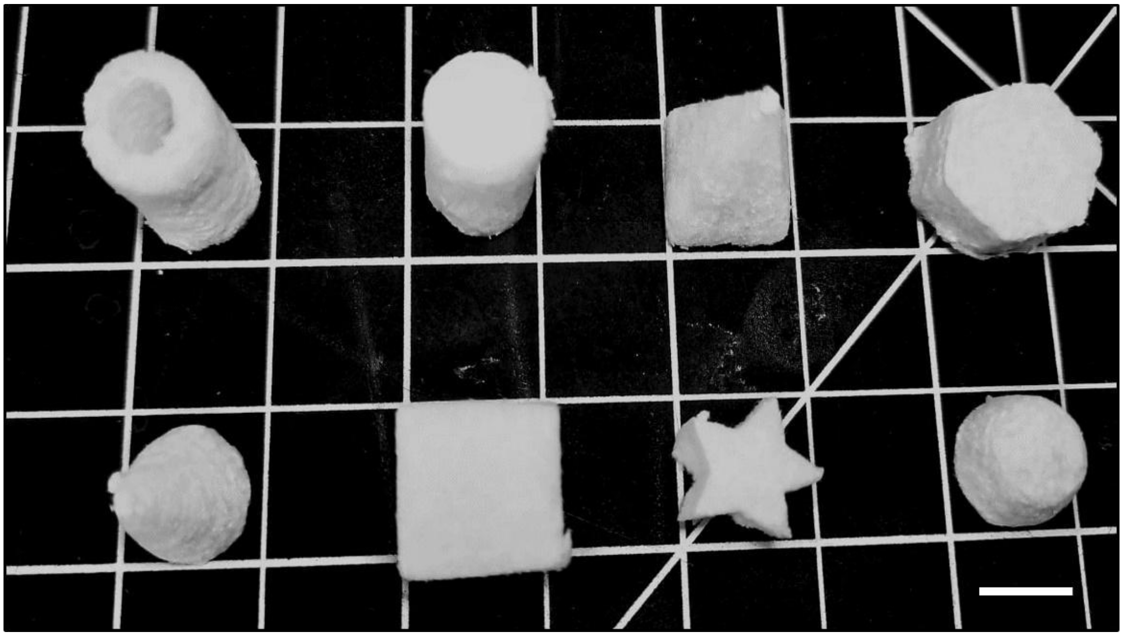

Figure S3 | Photographs showing hybrid aerogels (NF/MF-A1) with various customized shapes. NF/MF-A1: hybrid aerogels containing NF and MF at a ratio of 50:50 (w/w). Scale bar = 1 cm.

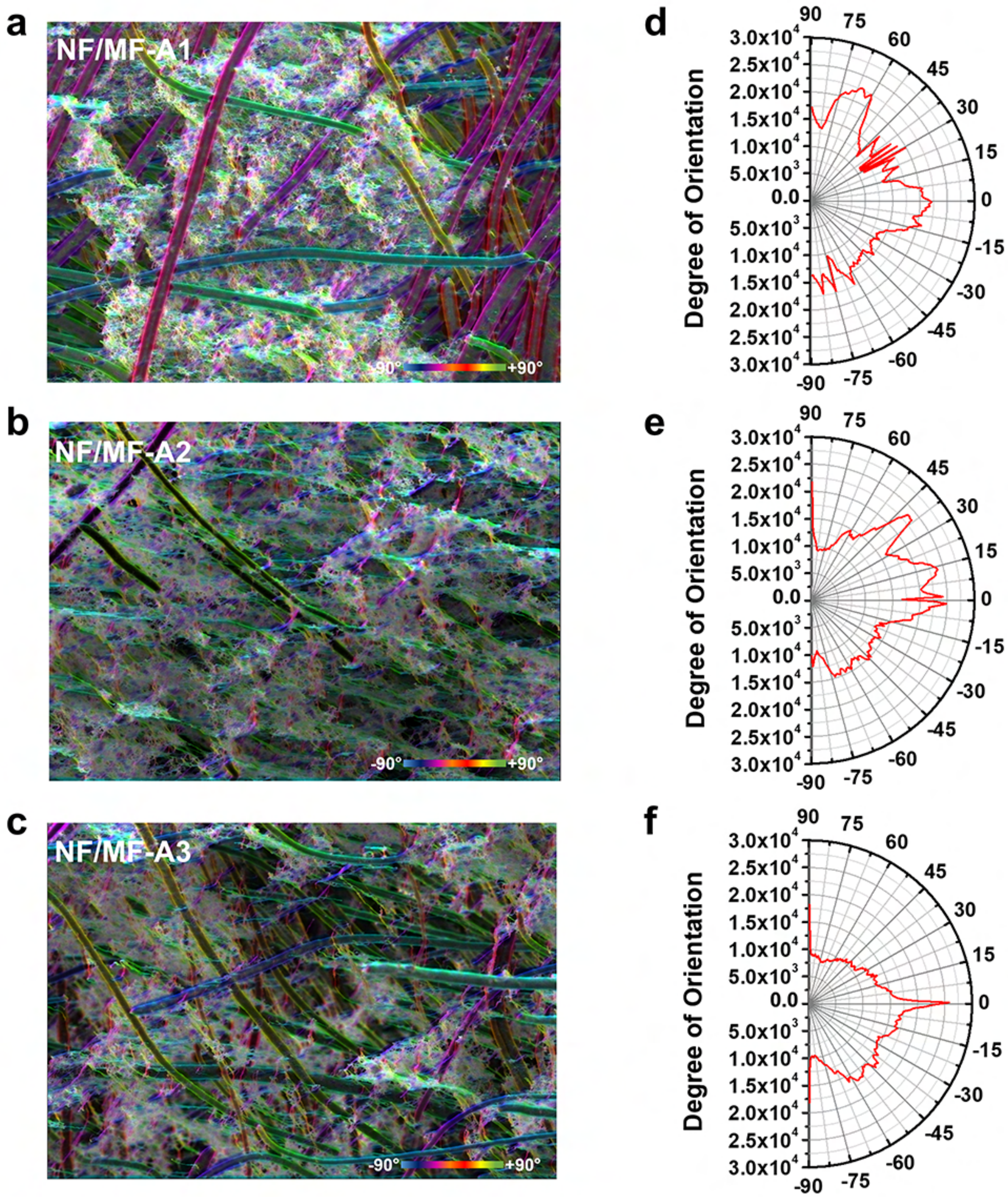

Figure S4 | Fiber orientations in hybrid aerogels. a-c, The angle mapping images of fiber orientations in NF/MF-A1, NF/MF-A2, and NF/MF-A3. **d-f**, Statistical histograms of fiber orientations in NF/MF-A1, NF/MF-A2, and NF/MF-A3. NF/MF-A1: hybrid aerogels containing NF/MF at a ratio of 50:50 (w/w). NF/MF-A2: hybrid aerogels containing NF/MF at a ratio of 25:75 (w/w). NF/MF-A3: hybrid aerogels containing NF/MF at a ratio of 75:25 (w/w).

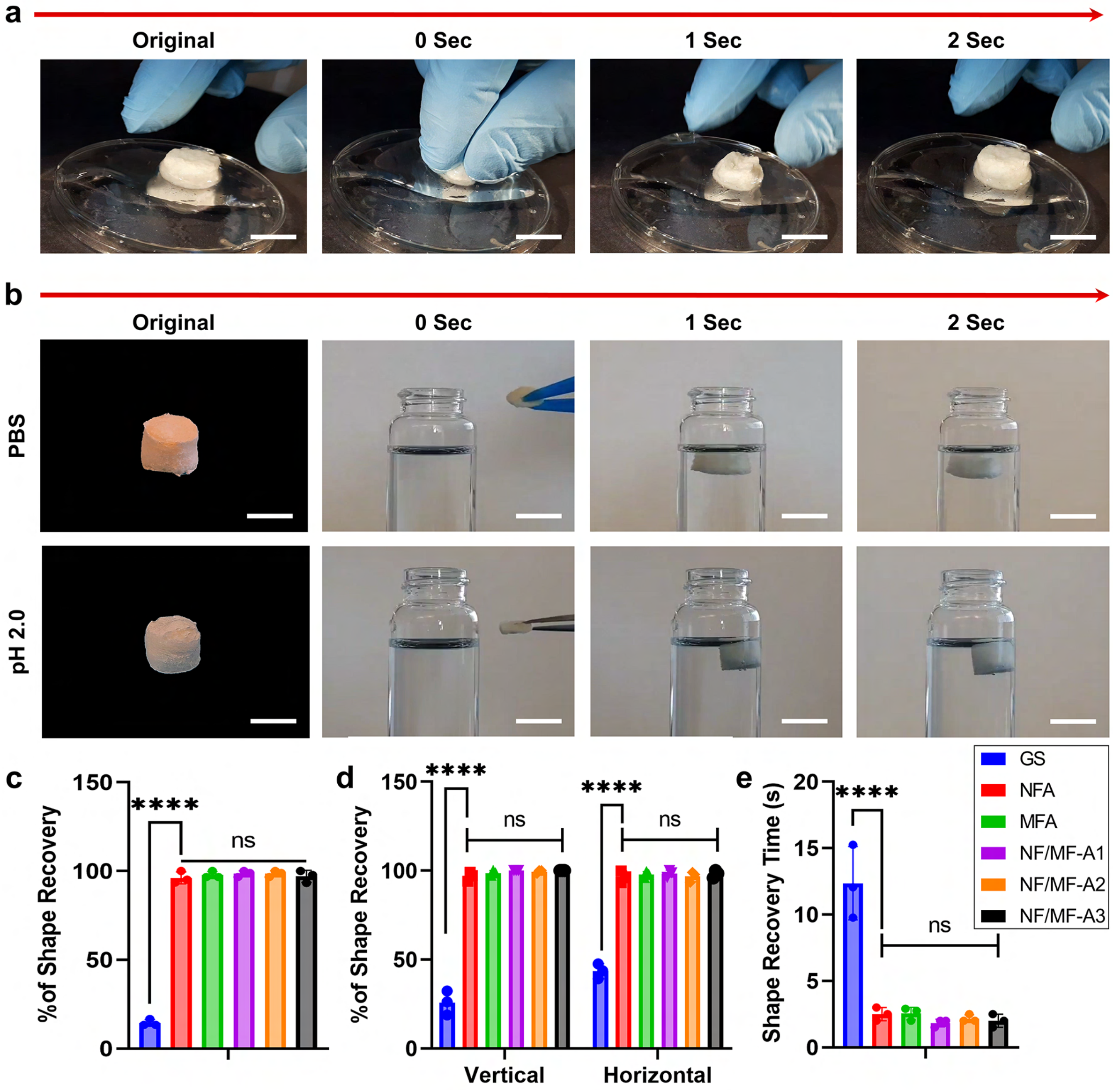

Figure S5 | Shape-recovery properties of hybrid aerogels. **a**, Photos showing the top-bending resistance of NF/MF-A1. **b**, Photos showing shape recovery of NF/MF-A1 in solutions with neutral (top panel) and acidic (lower panel) pHs. **c**, Percentages of shape recovery of tested samples in pH 2.0 after compressing the aerogels in vertical directions. **d**, Percentages of shape recovery in PBS after compressing the aerogels in horizontal and vertical directions. **e**, Shape recovery time. Data in c-e are presented as mean ± SD, $N = 3$. The significant difference was detected by one-way ANOVA with Tukey's multiple comparisons test. The 'ns' indicated no significant difference, ****$P < 0.0001$. GS: gelatin sponge; NF/MF-A1: hybrid aerogels containing NF/MF at a ratio of 50:50 (w/w). NF/MF-A2: hybrid aerogels containing NF/MF at a ratio of 25:75 (w/w). NF/MF-A3: hybrid aerogels containing NF/MF at a ratio of 75:25 (w/w). Scale bar = 1 cm.

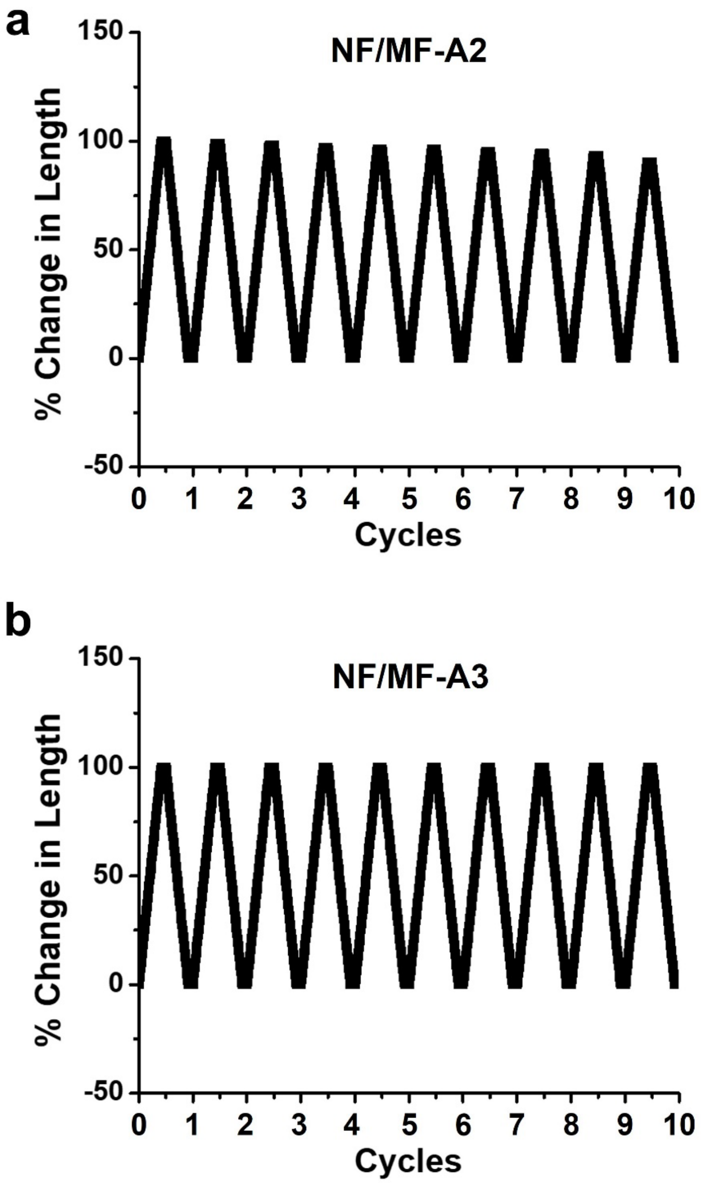

Figure S6 | a,b, Changes in length of NF/MF-A2 and NF/MF-A3 during 10 cycles of compression-relaxation test at 90% compressive strain. NF/MF-A2: hybrid aerogels containing NF/MF at a ratio of 25:75 (w/w). NF/MF-A3: hybrid aerogels containing NF/MF at a ratio of 75:25 (w/w).

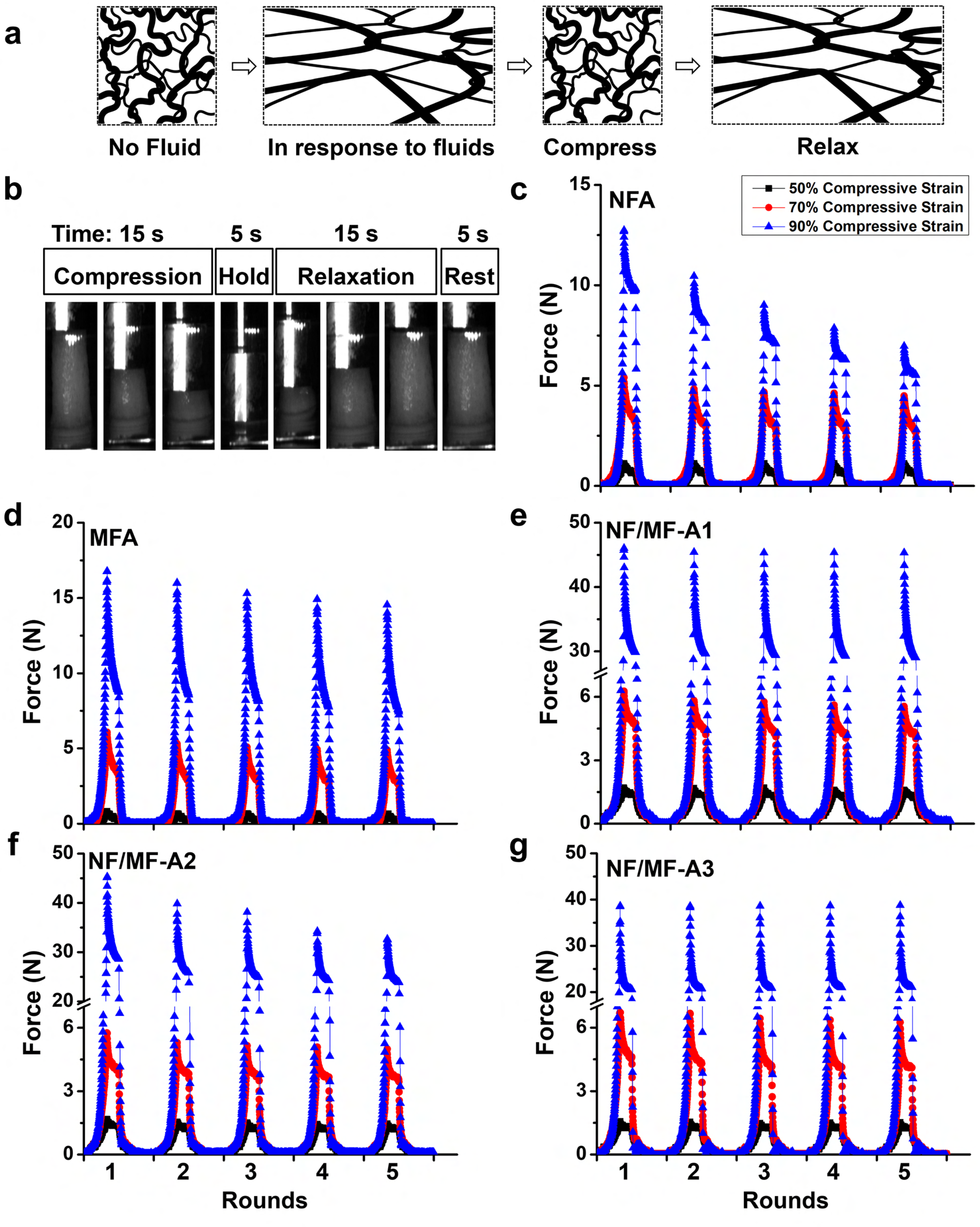

Figure S7 | Mechanical properties of hybrid aerogels. a, Schematics illustrate the fluid responsive shape-memory characteristics of hybrid aerogels. **b**, Photograph showing the experimental setup for each cycle. **c-g**, Cyclic compression test of NFA, MFA, NF/MF-A1,

NF/MF-A2, and NF/MF-A3 under 50%, 70%, and 90% compressive strains. NFA: nanofiber aerogels. MFA: microfiber aerogels. NF/MF-A1: hybrid aerogels containing NF/MF at a ratio of 50:50 (w/w). NF/MF-A2: hybrid aerogels containing NF/MF at a ratio of 25:75 (w/w). NF/MF-A3: hybrid aerogels containing NF/MF at a ratio of 75:25 (w/w).

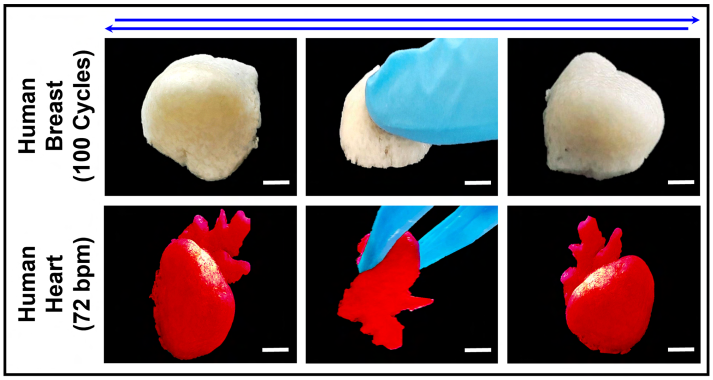

Figure S8 | Shape recoverable heart- and breast-shaped hybrid aerogels. Scale bar = 1 cm.

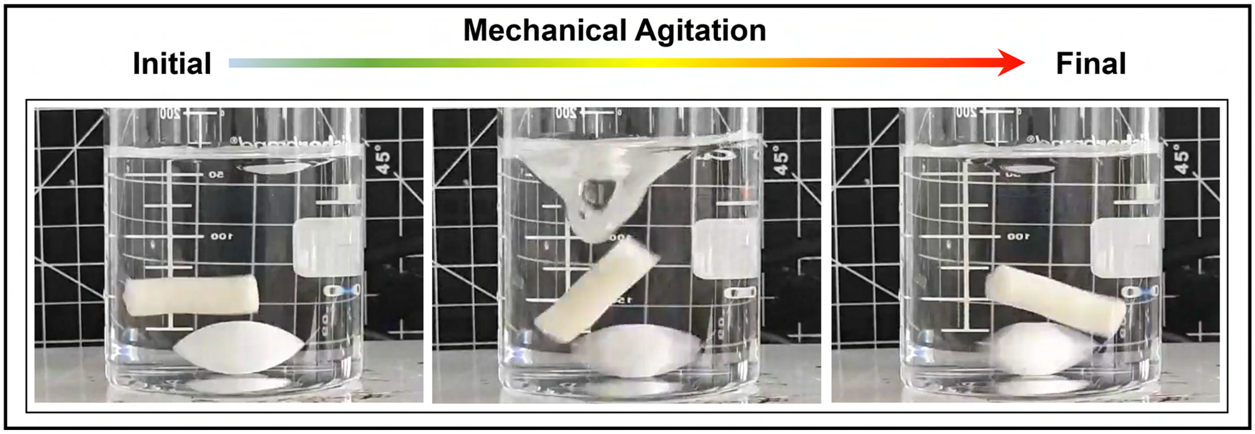

Figure S9 | Photographs illustrating that NF/MF-A1 maintains intact under mechanical agitations. NF/MF-A1: hybrid aerogels containing NF/MF at a ratio of 50:50 (w/w).

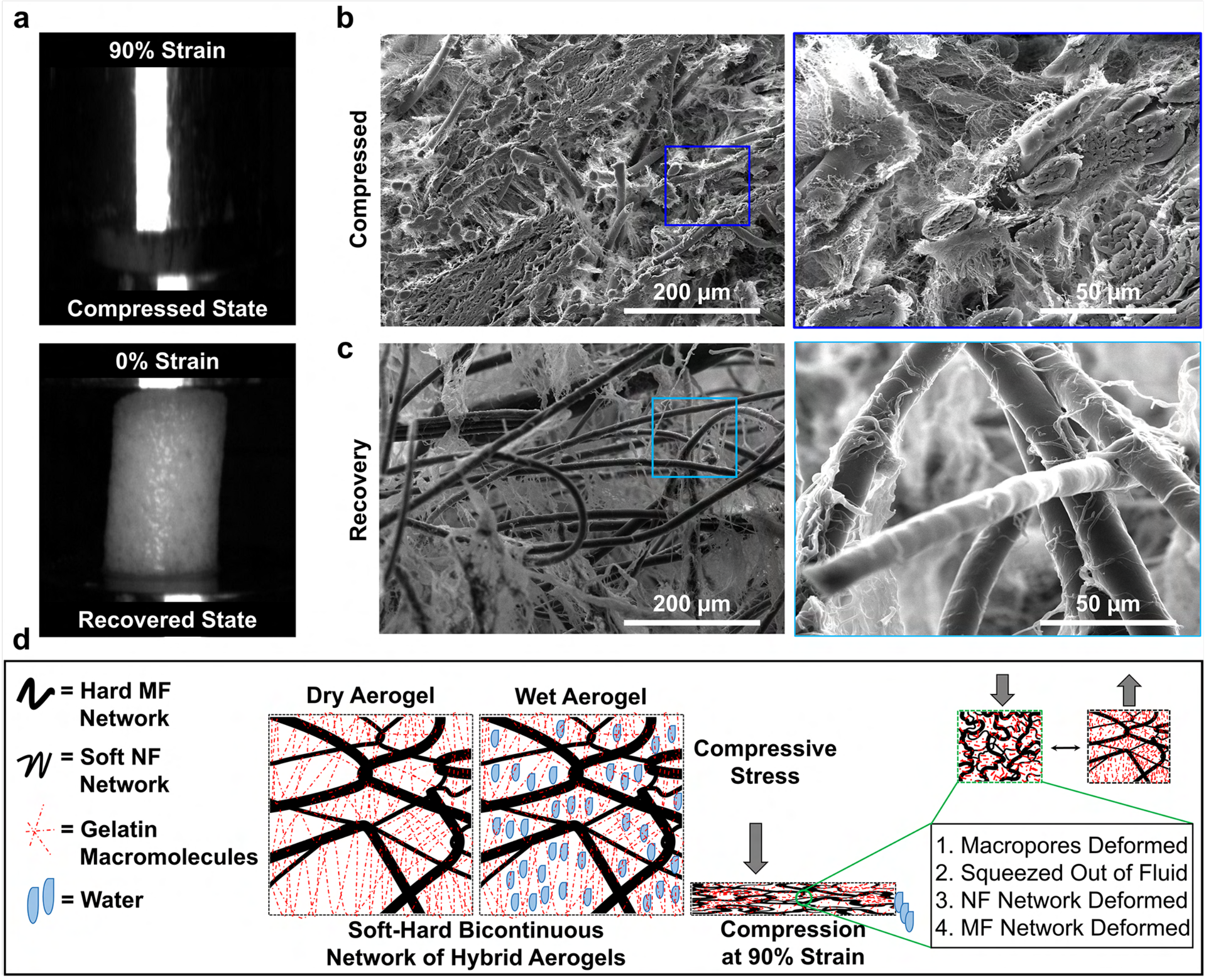

Figure S10 | a, Photos of hybrid aerogels under 90% and 0% compressive strain. **b**,**c**, SEM images showing the microstructure of the hybrid aerogels (NF/MF-A1) after compression and shape recovery. **d**, The proposed mechanism of high resilience of NF/MF-A1 hybrid aerogels under 90% compressive strain. NF/MF-A1: hybrid aerogels containing NF/MF at a ratio of 50:50 (w/w).

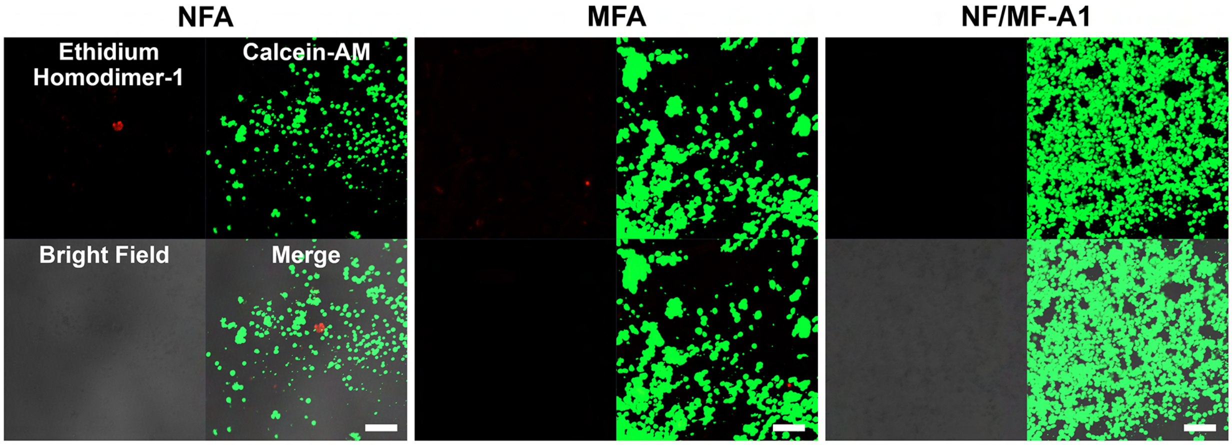

Figure S11 | Skin cell culture on hybrid aerogels. CLSM images showing live (green) /dead (red) staining of HaCaT cells cultured on different cylindrical aerogels (diameter = 8 mm, height = 2 mm) for 36 h. NFA: nanofiber aerogels. MFA: microfiber aerogels. NF/MF-A1: hybrid aerogels containing NF/MF at a ratio of 50:50 (w/w). Scale bar =100 µm.

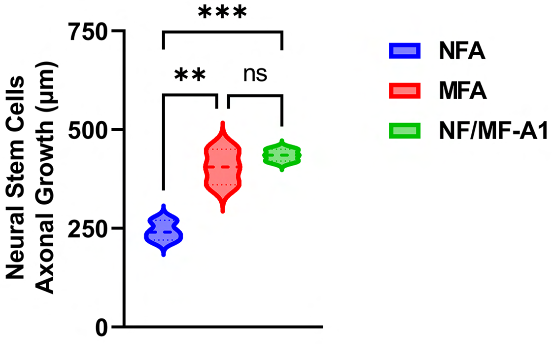

Figure S12 | Neural stem cell culture on hybrid aerogels. Migration of neural stem cells and axonal growth in the aerogels. Data are presented as mean ± SD, $N = 3$. The significant difference was detected by one-way ANOVA with Tukey's multiple comparisons test (**$p = 0.0017$, ***$p = 0.0007$, ns = a non-significant value of > 0.05). NFA: nanofiber aerogels. MFA: microfiber aerogels. NF/MF-A1: hybrid aerogels containing NF/MF at a ratio of 50:50 (w/w).

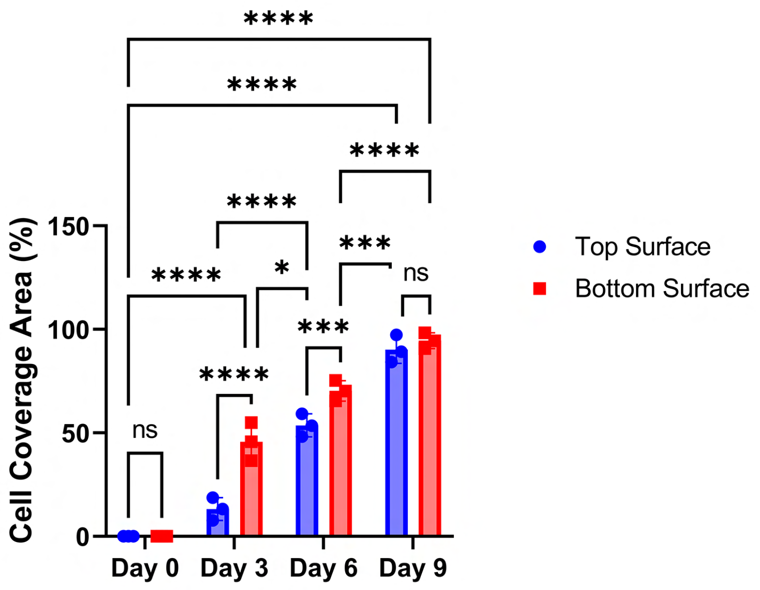

Figure S13 | Dermal fibroblast migration through NF/MF-A1. Migration of GFP-labeled dermal fibroblasts from the bottom surface to top surface of NF/MF-A1 at day 0, 3, 6, and 9. Data are presented as mean ± SD, $N = 3$. The significant difference was detected by two-way ANOVA with Tukey's multiple comparisons test ($n = 3$, *$p < 0.01$, ***$p \leq 0.0005$, ****$p < 0.0001$, ns = a non-significant value of > 0.05). NF/MF-A1: hybrid aerogels containing NF/MF at a ratio of 50:50 (w/w).

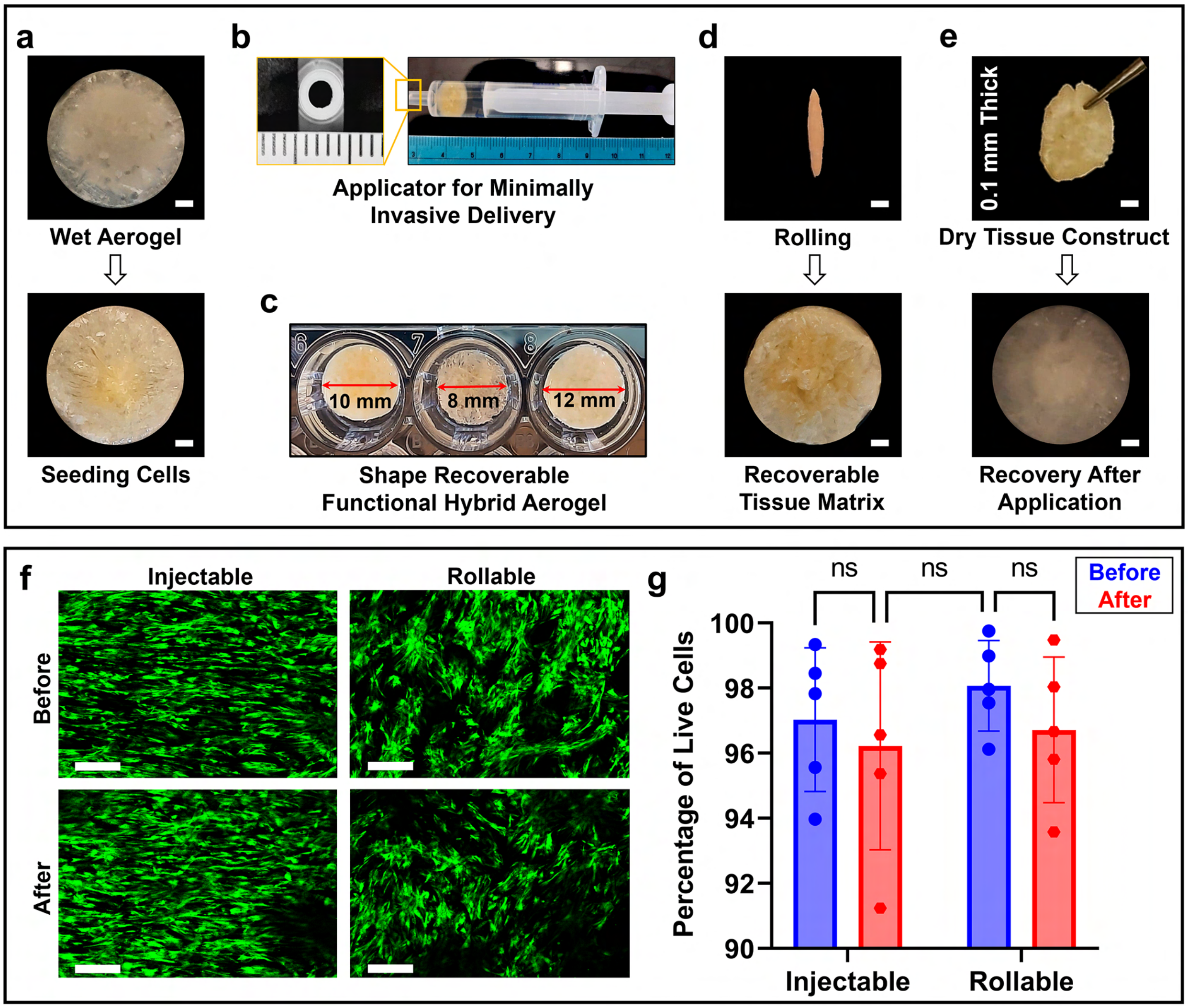

Figure S14 | Minimally invasive delivery of shape-recoverable hybrid aerogels. a, Sterilized aerogels (diameter = 10 mm and height = 2 mm) were immersed into cell culture media and GFP-labeled dermal fibroblasts were then seeded on aerogels. **b**, Applicator with a 1.7 mm inner diameter cannula for minimally invasive delivery. **c**, Photographs showing cell-containing aerogels with different sizes (diameters = 8, 10, 12 mm) can be delivered in a minimally invasive manner. **d**, Rolled tissue matrix before and after delivery. **e**, Dry decellularized aerogels before and after delivery. Scale bar = 2 mm. **f**, Confocal images showing GFP-labeled dermal fibroblasts in the cross-sectional area of NF/MF-A1 before and after injection. Scale bar = 100 μm. **g**, Percentages of viable cells present within NF/MF-A1 before and after injections. Data are presented as mean ± SD, $N = 5$. The significant difference was detected by two-way ANOVA with Tukey's multiple comparisons test (ns = a non-significant value of > 0.05).

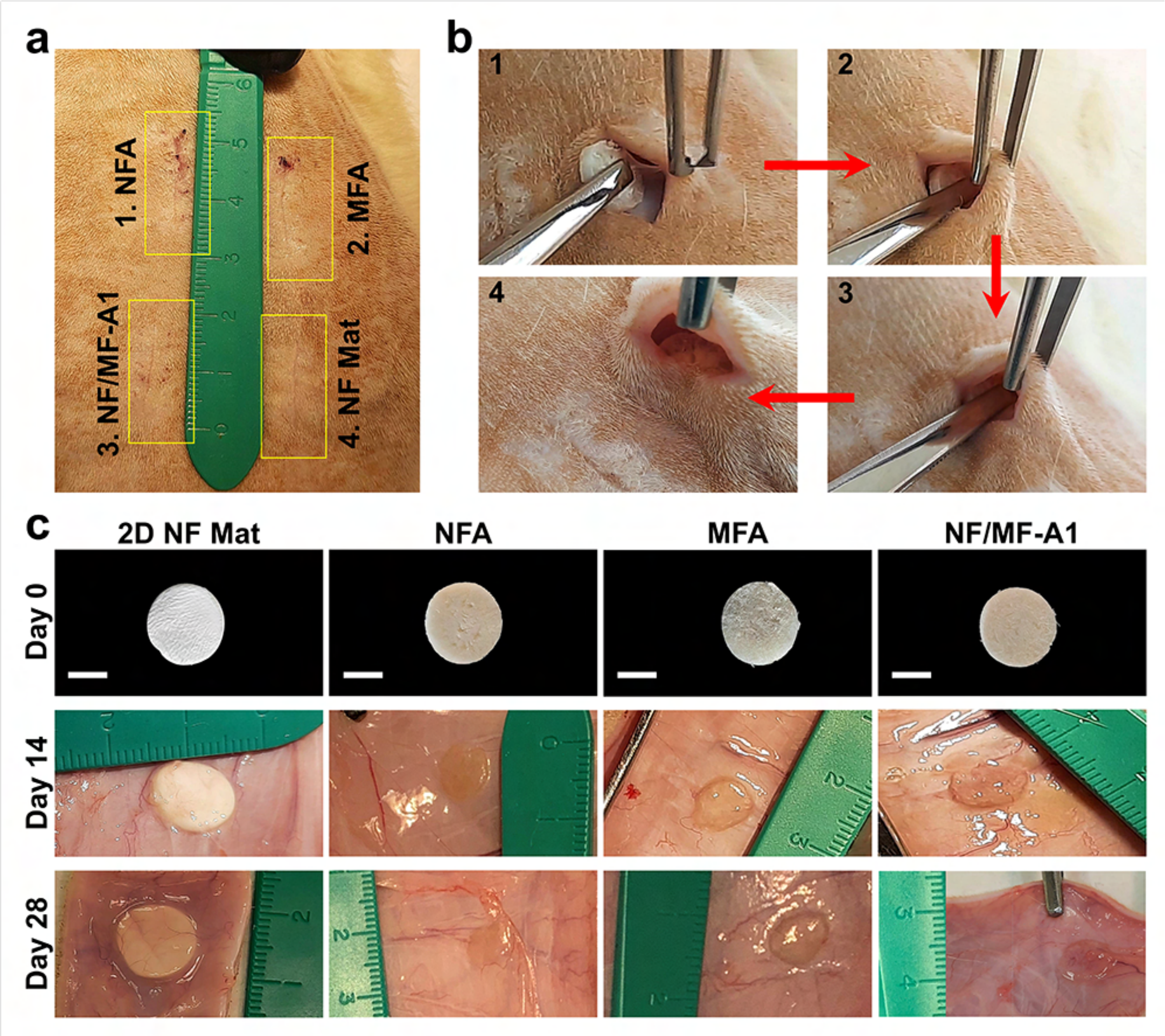

Figure S15 | The subcutaneous implantation of different samples to rats. a, Subcutaneous pockets were made through 1.5 cm incisions at four supraspinal sites on the dorsum. **b**, Photographs showing the procedure of subcutaneous implantation. **c**, Photographs showing different samples before implantation (Day 0) and after implantation (Day 14 and Day 28). N=12. Scale bar = 5 mm.

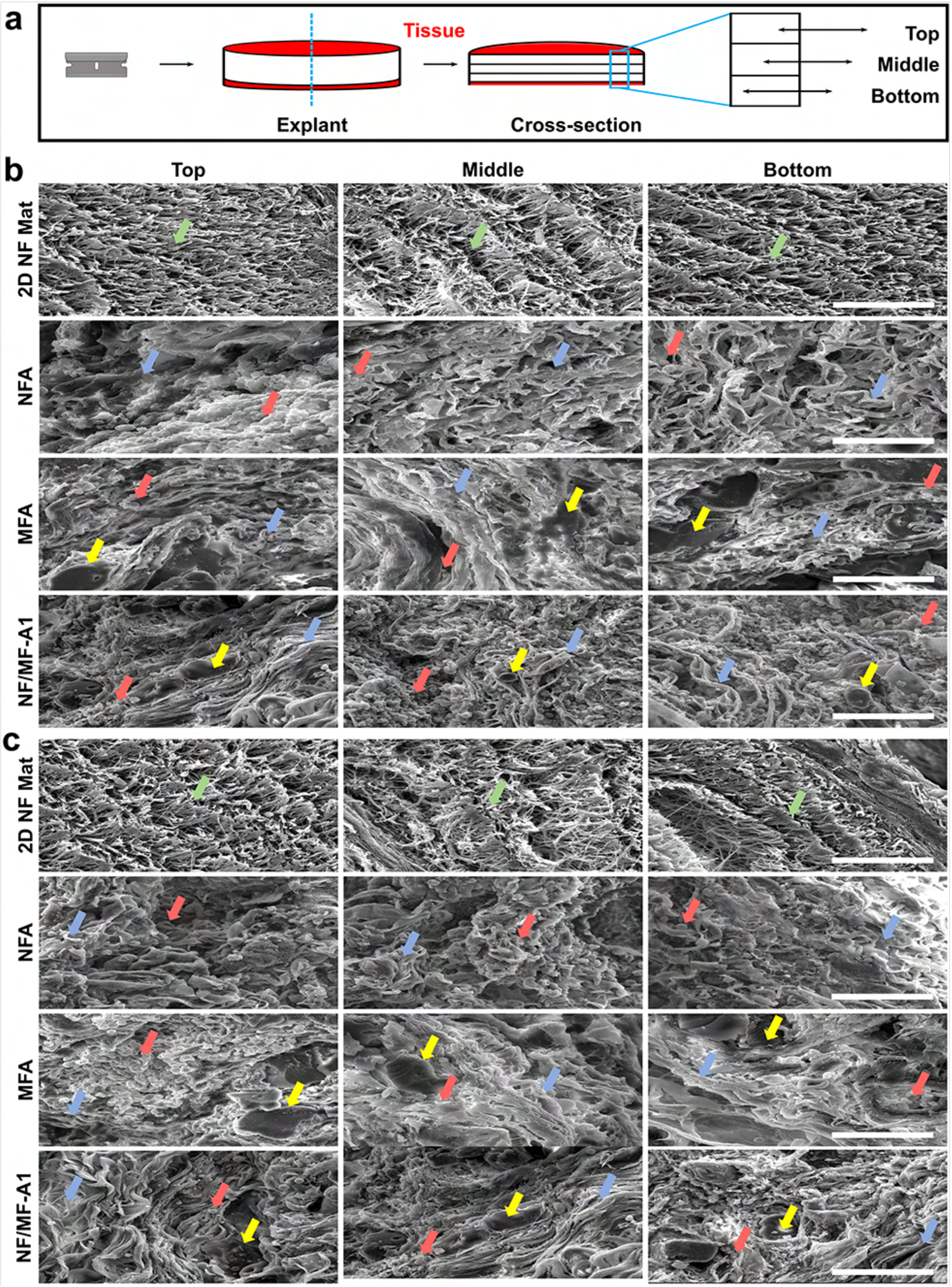
a
Tissue
Explant
Cross-section
Top
Middle
Bottom
b
Top
Middle
Bottom
2D NF Mat
NFA
MFA
NF/MF-A1
c
2D NF Mat
NFA
MFA
NF/MF-A1

Figure S16 | Morphological characterizations of explants. a, Schematic illustrating SEM imaging of cross sections of explants. The schematic was created in BioRender.com. **b**,**c**, SEM images showing cross sections of each explant after subcutaneous implantation for 14 and 28 days to rats. Green, yellow, blue, and red arrows indicate nanofibers (NFs), microfibers (MFs), deposited extracellular matrix (ECM), and blood cells. Scale bar = 100 μm.

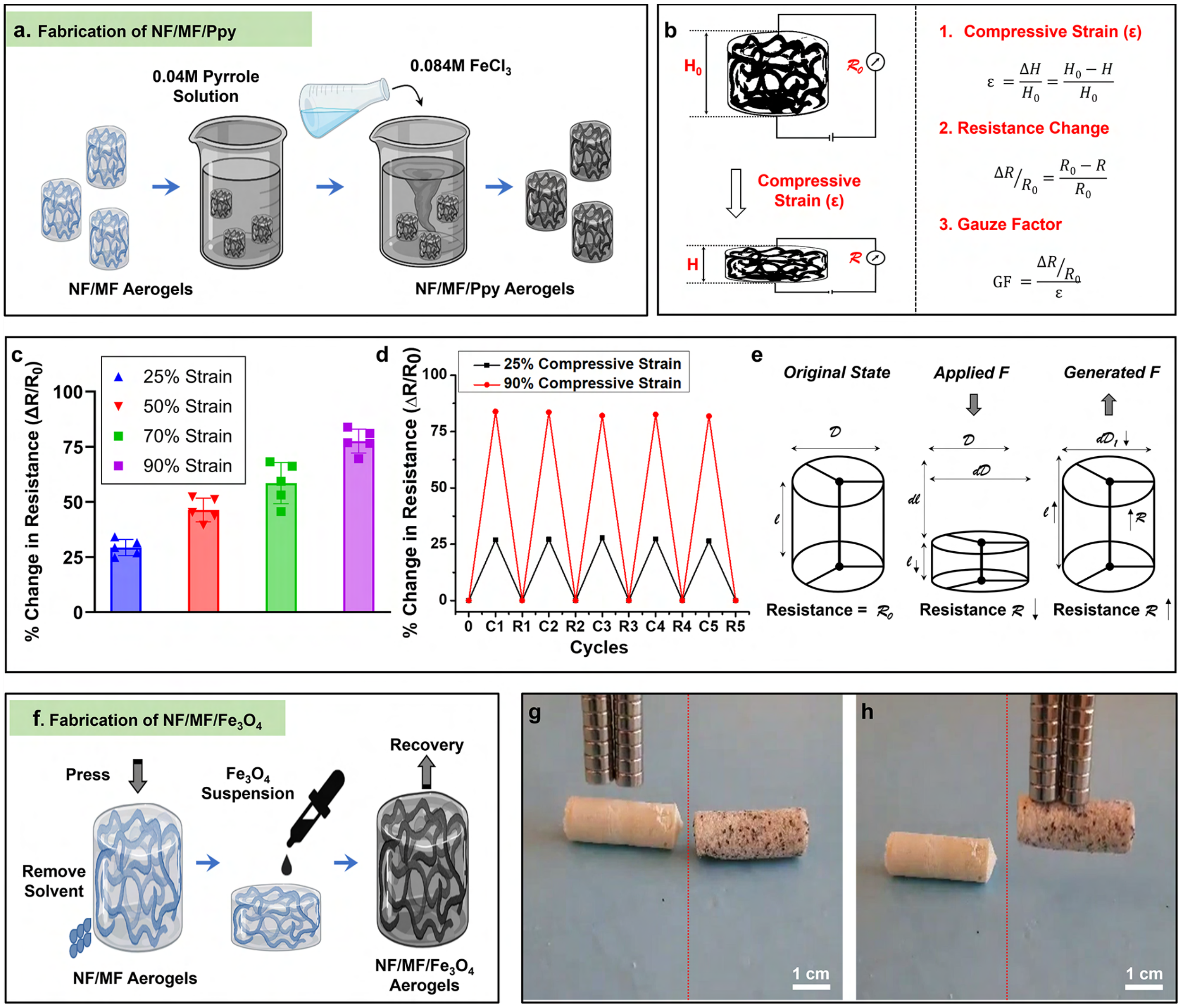

Figure S17 | Electrically conductive and magnetic field responsive hybrid aerogels. a, Schematic illustrating the fabrication of electrically conductive aerogels. **b**, Schematic illustrating the effect of compressive strain on the electrical resistance (left panel) and the related equations (right panel). R_0 and R represent the incipient resistance and momentary resistance. H_0 and H represent the original height and the height after compression. **c**, Change in resistance at different compressive strains. Data are presented as mean value +/- SD, $N = 5$. **d**, The cyclic change in electrical resistance at 25% and 90% compressive strains. **e**, Schematic illustrating the resistance value of conductive hybrid aerogels changes according to the different degrees of compression. **f**, Schematic illustrating the fabrication of hybrid aerogels with incorporation of Fe_3O_4 nanoparticles. **g**,**h**, Photographs showing NF/MF-A1 (left) fails to respond to magnetic field and the hybrid aerogel (right) after incorporation of Fe_3O_4 nanoparticles is responsive to a magnetic field. The schematics in a, b, e, and f created in BioRender.com.

References

1. Liu, L. *et al.* High axial ratio nanochitins for ultrastrong and shape-recoverable hydrogels and cryogels *via* ice templating. *ACS Nano* **13**, 2927–2935 (2019).
2. Jähne, B. *Digital Image Processing: Concepts, Algorithms, and Scientific Applications*. (Springer Berlin Heidelberg, 1995). doi:10.1007/978-3-662-03174-2.
3. Du, X. *et al.* Microchannelled alkylated chitosan sponge to treat noncompressible hemorrhages and facilitate wound healing. *Nat. Commun.* **12**, 4733 (2021).
4. McCarthy, A. *et al.* Extracellular matrix secretion mechanically reinforces interlocking interfaces. *Adv. Mater.* **35**, 2207335 (2023).
5. Zhang, Y. *et al.* An injectable antibacterial chitosan-based cryogel with high absorbency and rapid shape recovery for noncompressible hemorrhage and wound healing. *Biomaterials* **285**, 121546 (2022).
6. Wang, J., Lin, J., Chen, L., Deng, L. & Cui, W. Endogenous electric-field-coupled electrospun short fiber via collecting wound exudation. *Adv. Mater.* **34**, 2108325 (2022).
7. Sun, T. L. *et al.* Physical hydrogels composed of polyampholytes demonstrate high toughness and viscoelasticity. *Nat. Mater.* **12**, 932–937 (2013).
8. PRICE, G. R. Extension of covariance selection mathematics. *Ann. Hum. Genet.* **35**, 485–490 (1972).
9. Chen, S., McCarthy, A., John, J. V., Su, Y. & Xie, J. Converting 2D nanofiber membranes to 3D hierarchical assemblies with structural and compositional gradients regulates cell behavior. *Adv. Mater.* **32**, 2003754 (2020).
10. John, J. V. *et al.* Nanofiber Aerogels with precision macrochannels and LL-37-mimic peptides synergistically promote diabetic wound healing. *Adv. Funct. Mater.* **33**, 2206936 (2023).
11. Chen, Y., Yu, Q. & Xu, C.-B. A convenient method for quantifying collagen fibers in atherosclerotic lesions by ImageJ software. *Int. J. Clin. Exp. Med.* **10**, 14904–14910 (2017).
12. Chen, S. *et al.* Fast transformation of 2D nanofiber membranes into pre-molded 3D scaffolds with biomimetic and oriented porous structure for biomedical applications. *Appl. Phys. Rev.* **7**, 021406 (2020).
