# Supplementary material for: Mechanically resilient hybrid aerogels containing fibers of dual-scale sizes and knotty networks for tissue regeneration
Source: Nat Commun. 2024 Feb 5;15:1080. doi: 10.1038/s41467-024-45458-x (PMC10844217; doi:10.1038/s41467-024-45458-x)
Supplement: Supplementary file 3 — Description of Additional Supplementary Files [file 41467_2024_45458_MOESM3_ESM.pdf]

Description of Additional Supplementary Files

Title: Supplementary Movie 1

Description: Video of static electricity of NF/MF-A1.

Title: Supplementary Movie 2

Description: Video of mechanical strength, flexibility, and compression resistance capability.

Title: Supplementary Movie 3

Description: Video of top-bending resistance capability.

Title: Supplementary Movie 4

Description: Video of fast resilience of hybrid scaffolds at different pHs.

Title: Supplementary Movie 5

Description: Video of fast resilience and compression resistance capability of human breast shaped hybrid aerogels (NF/MF-A1).

Title: Supplementary Movie 6

Description: Video of cyclic compression and relaxation of human heart-shaped hybrid aerogels (NF/MF-A1) for 72 cycles/min.

Title: Supplementary Movie 7

Description: Video of stability analysis of NF/MF-A1 under strong mechanical agitation.

Title: Supplementary Movie 8

Description: Video of live (green) /dead (red) staining of HaCaT cells cultured on different cylindrical aerogels for 36 h.

Title: Supplementary Movie 9

Description: Video of minimally invasive delivery of cell-containing hybrid aerogels (NF/MFA1).

Title: Supplementary Movie 10

Description: Video of rolling and expansion of tissue matrix made of hybrid aerogels (NF/MFA1).

Title: Supplementary Movie 11

Description: Video of shape recovery of dry decellularized hybrid aerogels (NF/MF-A1).

Title: Supplementary Movie 12

Description: Video of hybrid aerogels as pressure sensors.

Title: Supplementary Movie 13

Description: Video of the magnetic responsive behavior of NF/MF-A1 functionalized with Fe3O4 nanoparticles.
